# Hepatic fibrosis: a manifestation of the liver disease evolution in patients with Ataxia-telangiectasia

**DOI:** 10.1186/s13023-023-02720-7

**Published:** 2023-05-05

**Authors:** Talita Lemos Neves Barreto, Roberto José de Carvalho Filho, David Carlos Shigueoka, Fernando Luiz Affonso Fonseca, Ariel Cordeiro Ferreira, Cristiane Kochi, Carolina Sanchez Aranda, Roseli Oselka Saccardo Sarni

**Affiliations:** 1 Department of Pediatrics, Division of Allergy, Clinical Immunology and Rheumatology, Universidade Federal de São Paulo (UNIFESP), 731 Otonis St., Vila Clementino, São Paulo, SP Brazil; 2 Department of Gastroenterology, Universidade Federal de São Paulo (UNIFESP), 1570 Loefgren St., Vila Clementino, São Paulo, SP Brazil; 3 Department of Diagnostic Imaging, Universidade Federal de São Paulo (UNIFESP), 800 Napoleão de Barros St., Vila Clmementino, São Paulo, SP Brazil; 4 Centro Universitário FMABC, 821 Príncipe de Gales Av., Santo André, SP Brazil; 5 Faculdade de Ciências Médicas da Santa Casa de São Paulo (FCMSCSP), 61 Dr. Cesário Motta Jr. St., São Paulo, SP Brazil; 6 Centro Universitário Saúde FMABC, 821 Príncipe de Gales Av., Santo André, SP Brazil

**Keywords:** Ataxia-telangiectasia, Hepatic fibrosis, Image techniques by elasticity, Insulin resistance, Inflammation

## Abstract

**Background:**

Ataxia-telangiectasia (A-T) is a DNA repair disorder characterized by changes in several organs and systems. Advances in clinical protocols have resulted in increased survival of A-T patients, however disease progression is evident, mainly through metabolic and liver changes.

**Objective:**

To identify the frequency of significant hepatic fibrosis in A-T patients and to verify the association with metabolic alterations and degree of ataxia.

**Methods:**

This is a cross-sectional study that included 25 A-T patients aged 5 to 31 years. Anthropometric data, liver, inflammatory, lipid metabolism and glucose biomarkers (oral glucose tolerance test with insulin curve—OGTT) were collected. The Cooperative Ataxia Rating Scale was applied to assess the degree of ataxia. The following were calculated: Homeostasis Model Assessment—Insulin Resistance, Homeostasis Model Assessment—Adiponectin (HOMA-AD), Matsuda index, aspartate aminotransferase (AST): platelet ratio index, nonalcoholic fatty liver disease fibrosis score and BARD score. Liver ultrasonography and transient liver elastography by FibroScan^®^ were performed**.**

**Results:**

Significant hepatic fibrosis was observed in 5/25 (20%). Patients in the group with significant hepatic fibrosis were older (*p* < 0.001), had lower platelet count values (*p* = 0.027), serum albumin (*p* = 0.019), HDL-c (*p* = 0.013) and Matsuda index (*p* = 0.044); and high values of LDL-c (*p* = 0.049), AST (*p* = 0.001), alanine aminotransferase (*p* = 0.002), gamma-glutamyl transferase (*p* = 0.001), ferritin (*p* = 0.001), 120-min glycemia by OGTT (*p* = 0.049), HOMA-AD (*p* = 0.016) and degree of ataxia (*p* = 0.009).

**Conclusions:**

A non-invasive diagnosis of significant hepatic fibrosis was observed in 20% of A-T patients associated with changes in liver enzymes, ferritin, increased HOMA-AD, and the severity of ataxia in comparison with patients without hepatic fibrosis.

**Supplementary Information:**

The online version contains supplementary material available at 10.1186/s13023-023-02720-7.

## Background

Ataxia-telangiectasia (A-T) is a DNA repair disorder characterized by changes in several human body organs and systems including progressive cerebellar degeneration, immunological changes, recurrent sinopulmonary infections, increased risk of cancer, especially of lymphoid origin, delay in growth and pubertal development, insulin-resistant diabetes and, more recently described, chronic liver disease [1–5].

A-T is an autosomal recessive condition caused by pathogenic variants in the *ATM* (ataxia telangiectasia mutated) gene, located on chromosome 11q22-23, that cause failure in the functioning of the ATM protein, a serine/threonine kinase, responsible for maintaining genomic stability, recognizing/correcting errors in DNA duplication, and controlling cell cycle [6]. Chronic oxidative stress due to impaired function or absence of ATM protein explains most of the characteristic symptoms of the disease [7, 8].

Despite advances in the treatment and control of the disease, such as immunoglobulin replacement, vaccination against respiratory illnesses, and antibiotic therapy prophylaxis that resulted in a reduction in morbidity and mortality, liver injury has been increasingly recognized as an important driver of mortality in A-T patients, particularly when progressive elevations of gamma-glutamyl transferase (GGT), aspartate aminotransferase (AST), and alanine aminotransferase (ALT) are observed in older patients [5, 9–12]. Publications addressing liver disorders in A-T patients are scarce, mostly case reports. Histopathological findings include non-alcoholic steatohepatitis (NASH), liver cirrhosis and hepato-cellular carcinoma (HCC) [13–18].

Although histopathological examination is considered the gold standard for the evaluation of necroinflammation and fibrosis, there are proposals for the use of indices and scores derived from formulas with biomarkers and liver imaging as non-invasive alternatives to the histological assessment, a particularly interesting proposal for A-T patients, in which liver biopsy is usually a challenging procedure [19, 20]. These noninvasive methods have been recommended for the identification of hepatic fibrosis, with varied availability and accuracy and that allow longitudinal evaluation and monitoring the liver disease progression [21–25].

To our knowledge, there are no publications evaluating liver fibrosis by imaging techniques and biomarkers in A-T patients. Thus, the aim of this study was to identify the frequency of significant hepatic fibrosis and to verify the association with metabolic changes and the degree of ataxia in A-T patients.

## Methods

This is a cross-sectional study that included 25 A-T patients from both genders, aged between 5 and 31 years, who met the diagnostic clinical criteria of the European Society for Immunodeficiencies (ESID), under a multidisciplinary follow-up at the Division of Allergy, Clinical Immunology, Rheumatology of the Department of Pediatrics of Universidade Federal de São Paulo (UNIFESP/EPM) [26]. Individuals undergoing oncological treatment, using hepatotoxic drugs or those with hepatitis B or C were excluded.

The study was approved by UNIFESP Research Ethics Committee (0081/2018). All the caregivers of patients signed an informed consent to be enrolled in this study.

### Anthropometric and pubertal development assessment

Anthropometric measurements included weight, height, skinfold thickness (triceps, biceps, subscapular, and suprailiac), mid-upper arm circumference, and waist circumference (WC) [27, 28].

For nutritional status classification, body mass index (BMI)-for-age and height-for-age z-scores for children and adolescents were calculated. Adults were classified according to BMI [27, 29]. The sum of the skinfold thickness was used to estimate the total body fat percentage [30–33].

WC was classified as altered when the WC to height ratio (WHtR) was equal or higher than 0.5 [34, 35]. Mid-upper arm circumference combined to the triceps skinfold was used to estimate the mid-upper arm muscle circumference (MUAMC) [28, 33].

Pubertal stage development was self-assessed using the Tanner rating scale (Marshall & Tanner) [36].

### Neurological assessment

A validated version of the International Cooperative Ataxia Rating Scale (ICARS) was applied to evaluate ataxia severity in all patients by a skilled physical therapist. For the severity classification, the following cut-off points were adopted: mild ataxia (1 to 30 points), moderate ataxia (31 to 60 points) and severe ataxia (> 60 points) [37].

### Laboratory tests

**Fig. 1:**
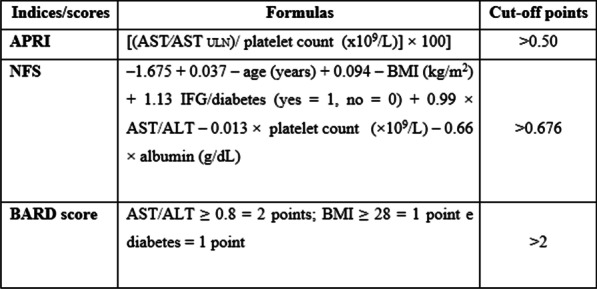
Indices/scores based on biomarkers for evaluation of hepatic fibrosis. *Abbreviations* APRI (aspartate aminotransferase to platelet ratio index); ULN (upper limit of normal); NFS (nonalcoholic fatty liver disease fibrosis score); BMI (body mass index); IFG (impaired fasting glucose); AST (aspartate aminotransferase), ALT (alanine aminotransferase) and BARD (B = BMI, AAR = AST/ALT ratio, D = Diabetes)

Liver injury biomarkers evaluated included ALT, AST, GGT, total proteins and fractions, alkaline phosphatase (ALKP), and alpha-fetoprotein (AFP), through standard methods. Cytokeratin-18 (CK-18) fragment levels were assessed by immune turbid metric assay (ELISA Kit. Elabscience Biotechnology Inc.^®^, USA). Indices and scores based on biomarkers for the evaluation of hepatic fibrosis were calculated. Figure 1 shows the formulas and cut-off points of the AST to platelet ratio index (APRI), nonalcoholic fatty liver disease fibrosis score (NFS), and BARD score [38–40].

Inflammation biomarkers evaluated included serum amyloid-A protein (SAA), tumor necrosis factor alpha (TNF-alpha), high-sensitivity C-reactive protein (hs-CRP), and ferritin, all measured by conventional methods. Adiponectin levels were assessed by immune turbid metric assays (ELISA Kit. Thermo Fisher Scientific Inc.^®^, Austria).

Lipid metabolism biomarkers included triglycerides (TG), total cholesterol (TC), low-density lipoprotein (LDL-c), high-density lipoprotein of cholesterol (HDL-c), and non-HDL-c cholesterol (NHDL-c). Cut-off points suggested by the American Academy of Pediatrics and the National Cholesterol Education Program (NCEP) were adopted for classification purposes [41, 42]. The NHDL-c was classified according to the criteria from the Bogalusa Heart Study and the NCEP Expert Panel [42, 43].

The oral glucose tolerance test (OGTT) was performed with insulin dosage. Blood samples for glycemic and insulin curves were obtained immediately after dextrose intake, in fasting and at 30, 60, 90 and 120-min intervals.

Glucose intolerance was considered when values between 140 and 199 mg/dL were found in 120 min. Diabetes was considered when fasting or at 120 min, values equal to or greater than 126 mg/dL and equal to or greater than 200 mg/dL, respectively [44].

Insulin resistance (IR) was assessed using the following indices: Homeostasis Model Assessment—Insulin Resistance (HOMA-IR), Homeostasis Model Assessment—Adiponectin (HOMA-AD) and Matsuda index that estimates whole-body insulin sensitivity [45–47]. For HOMA-AD classification, the cut-off points > 8.6 for children and > 14.3 for adolescents and adults were considered as diagnostic of IR [48].

### Liver imaging tests

#### Hepatic ultrasonography

Ultrasonography was performed for grading liver steatosis. The examination was performed by a single examiner (radiologist) using GE healthcare equipment, Logiq P6 model, 5 MHz multifrequency convex transducer. The changes in the liver parenchyma were stratified in the form of score (1–9): absent (0) when echotexture of the liver is normal, mild (1–3), moderate (4–6), and severe (7–9) diffuse steatosis [49].

#### Transient elastography

Hepatic transient elastography (TE) by FibroScan^®^ (Echosens, Paris, France) was performed by a single experienced examiner (hepatologist) for liver stiffness measurements (LSM, expressed in kPa), which offers an estimation of fibrosis staging, and controlled attenuation parameter evaluation (CAP, expressed in dB/m), which provides an estimate of the proportion of hepatocytes affected by steatosis [50–53].

After a 2-h-minimum fast, patients were put in dorsal decubitus, with the right arm raised to facilitate access to the right hepatic lobe. A M-size probe was positioned between the 9th and 11th intercostal spaces between the anterior and middle axillary lines. In each examination, to obtain at least 10 valid measurements, a maximum of 20 measurements were performed.

For reliability analysis of the TE examination, the following parameters were used: success rate (SR) which would be the equivalent of the quotient between the number of valid measurements and the total number of obtained measurements expressed in percentage and interquartile range to median of measurements ratio (IQR/Md).

The following reliability criteria were adopted: minimum of 10 valid measurements; SR ≥ 60%; IQR/Md < 30% for any liver stiffness value; and IQR/Md > 30% for liver stiffness values below 7.1 kPa [54]. TE results that did not meet these criteria were disregarded.

The non-invasive diagnosis of significant hepatic fibrosis was defined by the presence of LSM ≥ 7 kPa (cut-off point used for NAFLD) or a combination of an APRI score > 0.5 plus NFS > 0.675 and/or a BARD score ≥ 3 [21, 38–40].

The term “significant hepatic fibrosis” represents the presence of a substantial amount of extracellular matrix (mainly collagens), suggesting ongoing liver injury and high risk for progression towards cirrhosis and its complications. From a histological point of view, fibrous septa between portal tracts and central veins, with or without perisinusoidal/pericellular fibrosis, are usually considered as minimum criteria for diagnosing “significant liver fibrosis”, which also encompasses patients with bridging fibrosis and cirrhosis itself [55].

### Statistical analysis

Data were entered and consolidated in an Excel spreadsheet (Office Microsoft^®^) and analyzed using the statistical package SPSS 19.0 (IBM^®^). Categorical variables were presented as absolute and percentage values. Continuous variables were analyzed for normality using Shapiro–Wilk test. For comparisons between the two groups of patients, with or without significant liver fibrosis, the variables with parametric distribution were presented in the form of mean and standard deviation and compared by the independent t-Student test and the variables with non-parametric distribution were presented as median (minimum and maximum) and compared by the two-tailed Mann–Whitney U test. Spearman's correlation coefficient was used to assess the correlations. The statistical significance level of 5% (*p* < 0.05) was adopted.

Presence or absence of significant hepatic fibrosis (as assessed by LSM, APRI, NFS, and BARD scores) and presence or absence of liver steatosis (as assessed by liver ultrasound examination) were considered dependent variables. Age, ICARS score, variables related to nutritional status, liver, inflammatory, lipid, and glucose metabolism biomarkers were considered independent variables.

## Results

**Fig. 2:**
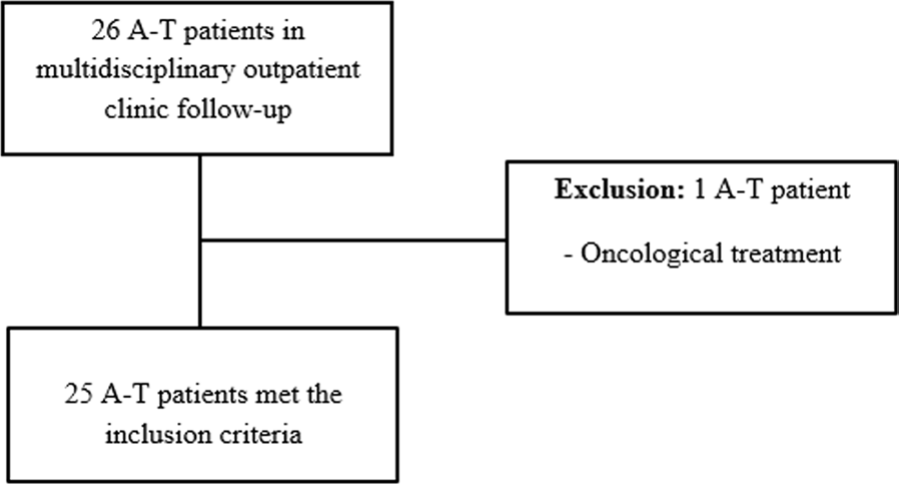
Flowchart of the study participants enrollment

The outpatient clinic currently follows 26 A-T patients and only one patient was excluded from the study for undergoing oncological treatment (Fig. 2). Mean age of A-T patients (*n* = 25) was 10.9 (± 3.8) years and 16/25 (64%) were males.

Most patients 13/25 (52%) were classified with moderate ataxia, 16/25 (64%) had dyslipidemia, 4/23 (17%) were diabetic (three diagnosed by OGTT performed in this study), 5/21 (23.8%) had IR according to HOMA-AD and 5/25 (20%) had values equal to or greater than twice the upper limit of normal for AST and ALT enzymes. Hepatic steatosis by ultrasonography was found in 13/20 (65%).

TE was performed on 22/25 (88%) of patients, and one LSM measurement was considered unreliable. Combining valid LSM measurements and the results of fibrosis scores (APRI, NFS, and BARD), significant liver fibrosis was non-invasively diagnosed in 5/25 (20%) of A-T patients. Table 1 shows the main characteristics of the cohort.

**Table 1:** Characteristics of the patients with Ataxia-telangiectasia

Variables		*N* (%)
Age (*n* = 25)	Children (< 10 years)	9 (36.0%)
	Adolescents (10 to 19 years)	11 (44.0%)
	Adults (≥ 20 years)	5 (20.0%)
Nutritional status (*n* = 25)	Underweight	8 (32.0%)
	Adequate	16 (64.0%)
	Overweight	1 (4.0%)
Fat mass (*n* = 24)	Low	4 (16.7%)
	Adequate	13 (54.1%)
	High	7 (29.1%)
MUAMC (*n* = 24)	Low	13 (54.1%)
	Adequate	11 (45.8%)
WHtR (*n* = 24)	Adequate	20 (83.3%)
	High	4 (16.7%)
Pubertal stage (*n* = 25)	Prepubertal	9 (36.0%)
	Pubertal	8 (32.0%)
	Postpubertal	8 (32.0%)
ICARS (*n* = 25)	Mild ataxia	3 (12.0%)
	Moderate ataxia	13 (52.0%)
	Severe ataxia	9 (36.0%)
hs-CRP (*n* = 25)	> 1 mg/L	7 (28.0%)
	≤ 1 mg/L	18 (72.0%)
Lipid profile (*n* = 25)	High total cholesterol	5 (20.0%)
	High LDL-c	5 (20.0%)
	High triglycerides	6 (24.0%)
	Low HDL-c	8 (32.0%)
	High NHDL-c	14 (56.0%)
Glucose metabolism (*n* = 23)	Diabetes	4 (17.4%)
	Insulin resistance** (*n* = 21)	5 (23.8%)
Hepatic steatosis (*n* = 20)	Absent	7 (35.0%)
	Mild	8 (40.0%)
	Moderate	5 (25.0%)
Significant liver fibrosis (*n* = 25)		5 (20.0%)
ALT (U/L) (*n* = 25)	≥ 2 xULN	4 (16.0%)
AST (U/L) (*n* = 25)	≥ 2 xULN	2 (8.0%)

*N (%)* absolute and percentage values

*MUAMC* mid-upper arm muscle circumference, *WHtR* waist circumference/height ratio, *hs-CRP* high sensitivity C reactive protein, *ICARS* International Cooperative Ataxia Rating Scale

^**^HOMA-AD Homeostasis Model Assessment- Adiponectin, *ALT* alanine aminotransferase, *AST* aspartate aminotransferase, *xULN* times the upper limit of normal

Four of the five A-T patients with non-invasive diagnosis of significant liver fibrosis underwent TE. Three presented moderate steatosis (grade 2), two had no steatosis, four were diabetic, three had IR, all had dyslipidemia, one was malnourished, and four had lean mass impairment. Table 2 shows the values of the parameters obtained by TE, as well as the fibrosis scores in patients considered to have *a non-invasive* diagnosis of significant liver fibrosis.

**Table 2:** Transient elastography parameters and fibrosis scores in patients with a non-invasive diagnosis of significant liver fibrosis

Variables	Patient 1	Patient 2	Patient 3	Patient 4	Patient 5*
Age (years)	20	29	31	27	29
Median CAP (dB/m)	364	198	306	209	NA
Median LSM (kPa)	8.9	38	8.7	11.8	NA
APRI	0.57	0.93	0.58	0.72	0.70
NFS	− 2.047	0.975	− 1.463	− 0.502	− 0.557
BARD score	2	3	1	0	3

*CAP* controlled attenuation parameter, *IQR* interquartile range, *LSM* liver stiffness measurement, *IQR/Md* interquartile range to median measurements ratio, *APRI* aspartate aminotransferase to platelet ratio index and *NFS* nonalcoholic fatty liver disease fibrosis score

^*^Transient elastography was not performed in patient 5. NA, not available

**Fig. 3:**
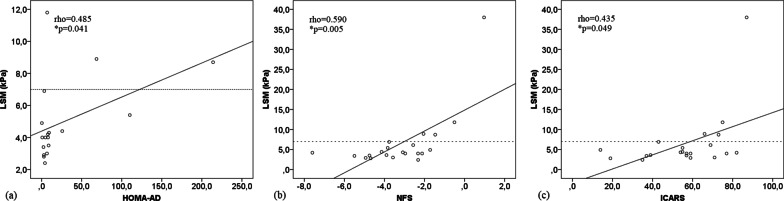
Scatter plot for the correlation of the liver stiffness measurement (LSM) values with the HOMA-AD (Homeostasis Model Assessment – Adiponectin) (**a**), nonalcoholic fatty liver disease fibrosis score (NFS) (**b**), and International Cooperative Ataxia Rating Scale (ICARS) (**c**). (*n* = 21). *Significance level of the Spearman correlation coefficient. Cut-off point ≥ 7 kPa adopted for diagnosing significant hepatic fibrosis (dotted line)

A significant and direct correlation was observed between LSM measurement obtained by TE and age (rho = 0.717; *p* < 0.001), GGT (rho = 0.578; *p* = 0.006), ICARS score (rho = 0.435; *p* = 0.049), NFS (rho = 0.590; *p* = 0.005), HOMA-AD (rho = 0.485; *p* = 0.041). LSM also exhibited a significant and indirect correlation with adiponectin level (rho = − 0.613; *p* = 0.003). Figure 3 shows the correlation between LSM measurement by TE and HOMA-AD, NFS, and ICARS score.

According to the presence or absence of non-invasive diagnosis of significant liver fibrosis, A-T patients were divided into two groups, which were compared for relevant clinical and laboratorial variables. Significant differences between groups were found regarding age (*p* < 0.001), MUAMC (*p* < 0.001), WC (*p* = 0.008), ICARS score (*p* = 0.009), platelets count (*p* = 0.027), albumin (*p* = 0.019), HDL-c (*p* = 0.013), LDL-c (*p* = 0.049), AST (*p* = 0.001), ALT (*p* = 0.002), GGT (*p* = 0.001), ferritin (*p* = 0.001), 120-min glycemia (*p* = 0.049), Matsuda index (*p* = 0.044) and HOMA-AD (*p* = 0.016) (Table 3).

**Table 3:** Bivariate analysis to identify variables associated with the presence of a non-invasive diagnosis of significant hepatic fibrosis in patients with Ataxia-telangiectasia

Variables	Unit	Absence of significant hepatic fibrosis (*n* = 20)	Presence of significant hepatic fibrosis (*n* = 5)	*p*-value^a,b^
Age	years	11.1 ± 4.2^c^	27.2 ± 4.3	** < 0.001**^**a**^
BMI	kg/m^2^	14.8 (11.8–26.8)	20.8 (10.6–24.1)	0.118^b^
Fat mass	%	17.9 ± 7.6	20.4 ± 5.4	0.553^a^
MUAMC	mm	169.8 ± 42.8	240.8 ± 6.9	** < 0.001**^**a**^
WC	cm	54.5 (47.0–90.0)	78.0 (68.0–82.0)	**0.008**^**b**^
WHtR	–	0.44 ± 0.06	0.49 ± 0.03	0.098^a^
ICARS	points	49.7 ± 20.1	76.0 ± 7.7	**0.009**^**a**^
Platelets count	10^3^/mm^3^	382 ± 100.5	266 ± 85.5	**0.027**^**a**^
Albumin	g/dL	4.5 ± 0.3	4.0 ± 0.7	**0.019**^**a**^
TC	mg/dL	167.9 (119.7–247.0)^d^	180.1 (172.4–289.5)	0.154^b^
LDL-c	mg/dL	101.7 (69.0–179.2)	130.0 (102.4–198.9)	**0.049**^**b**^
HDL-c	mg/dL	47,3 ± 12,6	30.9 ± 10.1	**0.013**^**a**^
TG	mg/dL	77.1 (35.0–196.7)	110.4 (53.4–331.0)	0.103^b^
NHDL-c	mg/dL	115.5 (76.0–189.0)	152.1 (131.0–265.1)	0.067^b^
AST	U/L	29.9 (16.6–74.3)	75.5 (46.9–82.3)	**0.001**^**b**^
ALT	U/L	20.7 (11.4–73.4)	89.4 (32.3–144.5)	**0.002**^**b**^
GGT	U/L	26.0 (7.0–102.0)	259.0 (108.0–612.0)	**0.001**^**b**^
ALKP	U/L	230.5 (113.0–497.2)	176.7 (111.2–355.0)	0.684^b^
CK-18	mIU/mL	252.8 (39.2–741.1)	191.4 (21.4–333.8)	0.374^b^
AFP	UL/mL	281.9 ± 177.8	240.4 ± 133.9	0.632^a^
Ferritin	ng/mL	82.3 (29.0–256.0)	419.0 (303.0–1538.0)	**0.001**^**b**^
hs-CRP	mg/L	1.0 (0.39–9.8)	2.2 (0.20–53.1)	0.424^b^
TNF-alpha	pg/mL	139.8 ± 8.5	142.0 ± 14.9	0.768^a^
Adiponectin	µg/mL	7.0 ± 3.9	3.5 ± 2.9	0.084^a^
SAA	ng/mL	11.3 (9.0–147.2)	20.4 (9.0–73.7)	0.111^b^
Fasting glucose	mg/dL	85.7 ± 9.3	90.7 ± 17.9	0.628^a^
120-min glycemia	mg/dL	90.2 ± 22.3	224.5 ± 84.8	**0.049**^**a**^
HOMA-IR	–	1.4 (0.08–4.5)	6.9 (1.4–20.9)	0.060^b^
HOMA-AD	–	4.1 (0.25–110.0)	108.1 (6.9–213.9)	**0.016**^**b**^
Matsuda index	–	9.2 (1.5–81.3)	3.3 (0,6–4.5)	**0.044**^**b**^

The bold text means there was statistical significance

^a^Significance Level of independent Student t-test

^b^Significance Level of the Mann–Whitney U test

^c^Mean (standard deviation)

^d^Median (minimum–maximum)

*BMI* body mass index, *MUAMC* mid-upper arm muscle circumference, *WC* waist circumference, *ICARS* International Cooperative Ataxia Rating Scale, *TC* Total cholesterol, *HDL-c* cholesterol high density lipoprotein, *LDL-c* cholesterol low density lipoprotein, *TG* Triglycerides, *NHDL-c* non-HDL-c, *ALT* alanine aminotransferase, *AST* aspartate aminotransferase, *GGT* gama-glutamyl transferase, *ALKP* alkaline phosphatase, *CK-18* cytokeratin-18, *AFP* alpha-fetoprotein, *hs-CRP* high sensitivity C reactive protein, *TNF-alpha* tumor necrosis factor alpha, *SAA* serum amyloid A protein, *min.* minutes, *HOMA-IR* Homeostasis Model Assessment—Insulin Resistance, *HOMA-AD* Homeostasis Model Assessment—Adiponectin

In calculating the accuracy of values equal to or greater than twice the upper limit of normal for AST and ALT enzymes as predictors of significant liver fibrosis, a sensitivity of 80% (CI95% 28.4–99.5) and a specificity of 95% (CI95% 75.1–99.9) were observed, which suggests that the longitudinal evaluation of these tests may contribute to the screening of progressive liver fibrosis in these patients during follow-up.

Liver biomarkers, inflammation and HOMA-IR, HOMA-AD and Matsuda index were compared between patients with and without liver steatosis as assessed by ultrasound, with no statistically significant difference being observed between groups for any of the variables (Additional file 1: Table S1).

**Fig. 4:**
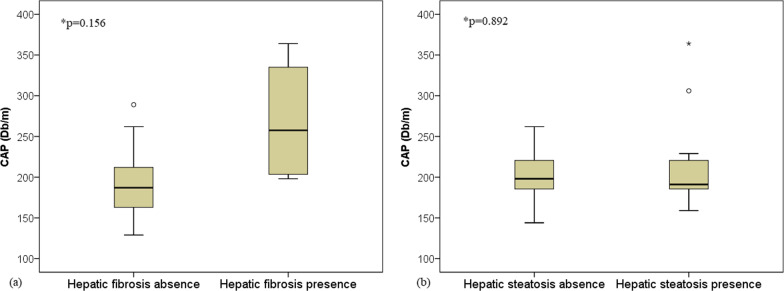
Median values of the controlled attenuation parameter (CAP) measurement between patients without (*n* = 17) and with significant hepatic fibrosis (*n* = 4). *Significance level by Student's t-test (**a**). Median CAP values between patients without (*n* = 7) and with ultrasound liver steatosis (*n* = 11). *Significance level of the Mann–Whitney U test (**b**)

Assessing the ability of the ultrasound assessment of steatosis for predicting the presence of significant hepatic fibrosis, a sensitivity of 60% (CI95% 14.7–94.7) and a specificity of 33% (CI95% 11.8–61.6) were observed. This finding suggests that the presence of hepatic steatosis on ultrasound does not seem to contribute to the screening of significant hepatic fibrosis in A-T patients. Likewise, there was no significant association between the degree of liver steatosis as assessed by CAP measurements and the non-invasive diagnosis of significant hepatic fibrosis, even if higher values have been identified in these patients (269.3 ± 79.7 dB/m vs. 194.7 ± 41.3 dB/m; *p* = 0.156). In addition, no significant difference was found for the CAP measurement between patients with and without liver steatosis on ultrasound (198 [144–262] dB/m vs. 191 [169–364] dB/m; *p* = 0.892) (Fig. 4).

APRI values were evaluated longitudinally in four A-T patients with significant hepatic fibrosis by TE. An increase in APRI values was observed over the years suggesting disease progression (Additional file 2: Fig. S1).

## Discussion

The present study showed that 20% of the A-T patients evaluated presented a non-invasive diagnosis of significant hepatic fibrosis. It is important to emphasize that all the patients with significant liver fibrosis had dyslipidemia and four had diabetes. The presence of meaningful fibrosis was associated with the severity of ataxia and with higher values of liver enzymes, ferritin, 120-min glycemia by OGTT and the HOMA-AD and lower values for the Matsuda index, compared to A-T patients without liver fibrosis.

Regarding the liver biomarkers evaluated, only liver enzymes (ALT, AST and GGT) were elevated in patients with a non-invasive diagnosis of significant hepatic fibrosis. Values equal to or greater than twice the upper limit of normal for AST and ALT enzymes as predictors of significant liver fibrosis revealed a sensitivity of 80% and a specificity of 95%. In a previous study performed by our group, elevated ALT and AST levels have been observed mainly during adolescence in A-T patients [5]. In a retrospective study with A-T patients conducted by Donath et al., a steady upward increase of ALT and GGT was observed, especially from the age of 12 [9]. In another retrospective study, elevation of liver enzymes was observed in younger A-T patients with a mean age of 9.97 ± 5.09 years, as well as a significant association with the presence of dyslipidemia [10]. Therefore, serial monitoring of AST and ALT levels from the age of 10 is highly recommended for the screening of liver fibrosis in A-T patients.

In the present study, patients diagnosed with diabetes had fasting glucose within the normal range and were only identified by OGTT with 120-min glycemia above 200 mg/dL. These findings suggest the importance of performing OGTT for early identification and treatment of diabetes, especially in adolescents. In another cohort of A-T patients, a progressive increase in glycated hemoglobin (HbA1c) and fasting glucose with advancing age were observed [56]. In that study, OGTT was considered to have good sensitivity for IR screening and HbA1c was recommended for assessing therapy response [56].

Epidemiological evidence suggests that diabetes is associated with a worse evolution of the liver disease and the use of hypoglycemic agents would be associated with a delay in the evolution of the disease. In NASH-associated fibrosis, the principal cell type responsible for extracellular matrix production is the hepatic stellate cell (HSC). Hyperglycemia seems to directly stimulate HSC, maintaining insulin resistance and, therefore, chronic inflammation, which could lead to worsening of the liver disease [57–60]. In addition, due to A-T, these patients are at a higher risk for the development of hepatocellular carcinoma.

The values of the HOMA-AD and the Matsuda index differed significantly between patients with and without significant hepatic fibrosis, fact not observed for HOMA-IR. A controlled study has also verified lower values of Matsuda index in A-T patients as compared to healthy individuals (5.96 6 ± 0.77 vs.11.03 ± 1.69; *p* = 0.019) and similar values of HOMA-IR between groups [61]. A recent study evaluating Brazilian children and adolescents has found a better performance of HOMA-AD for metabolic syndrome screening as compared to HOMA-IR [48]. Furthermore, Hung et al. observed that HOMA-AD appears to be sensitive for detecting small changes in insulin sensitivity in patients with or without diabetes [62]. Therefore, HOMA-IR does not seem to be the most appropriate method to identify IR in A-T patients.

Serum ferritin was the only inflammatory biomarker that had higher values in patients with significant hepatic fibrosis. Experimental study with the aim of investigating iron regulation, regulatory genes, and markers of oxidative stress in liver tissue of ATM-deficiency mice, described higher values of serum and hepatic iron, serum ferritin, and serum hepcidin when compared to controls. This study suggested that the increase in tissue iron would be associated with hepatic oxidative stress resulting from iron-induced increase in hepcidin, which can suppress its export by ferroportin, which is considered a protective mechanism in response to oxidative stress [63]. Therefore, it is suggested that the increase in serum ferritin may contribute to the chronic oxidative stress presented by A-T patients and, consequently, to the development of liver disease.

Another noteworthy fact was the significant and direct correlation between the median LSM obtained by TE and the ICARS score. In a retrospective study aimed at determining the evolution of liver disease and its relationship with age and neurological impairment in A-T patients, a significant and direct correlation was found between the Klockgether Ataxia Scale (KAS) score and age, and levels of AFP, GGT, and ALT [9]. In a recent study conducted by our group with A-T patients, significant and positive correlations were found between the severity of ataxia, age and metabolic changes, including impairment of liver damage markers and IR [64].

A-T patients with significant hepatic fibrosis presented with higher CAP values obtained by TE, as compared to those without significant hepatic fibrosis, although this difference has not achieved statistical significance. In addition, ultrasound examinations observed that three out of the five patients with significant hepatic fibrosis presented with steatosis. Therefore, only the simple identification of hepatic steatosis by both ultrasound and CAP was not related to the presence of a relevant amount of liver fibrosis in A-T patients, which suggests that the occurrence of steatosis per se is not a predictive factor for diagnosing fibrosis in these patients.

One of the mechanisms of ATM protein activation is attributed to the action of reactive oxygen species (ROS), resulting in increased concentrations of antioxidants and the repair of oxidative DNA damage. In the absence/deficiency of ATM, A-T patients have low antioxidant capacity and, as a result, macromolecules, lipids and DNA are exposed to constant oxidative stress and its detrimental consequences [8, 65, 66]. NAFLD and A-T share a similar pathogenic mechanism of ROS generation and mitochondrial dysfunction that contributes to the development of lesions [7]. Thus, it is postulated that oxidative stress has a relevant contribution in the genesis of liver disease associated with A-T.

The mechanisms involved in the evolution of NAFLD are not fully understood. Changes in proteins such as ATM could play a role in these mechanisms since this protein is associated with DNA integrity and mitochondrial homeostasis. An experimental study showed that ATM-deficient mice presented a reduction and delay in DNA replication during liver regeneration. Furthermore, when partial hepatectomy was performed, an increase in apoptosis was observed, which indicates that the ATM protein is involved in the regeneration and survival of hepatocytes [67].

A recent study that analyzed the expression of messenger RNAs, total proteins, or phosphoproteins related to the ATM pathway of individuals with healthy liver, hepatic steatosis, and NASH, found a causal association between the ATM pathway and NAFLD. During the steatosis phase, there was low ATM activation, which caused mitochondrial dysregulation and greater DNA damage, in addition to reduced growth of the hepatocyte. In NASH, there was greater ATM depletion, with a greater degree of DNA damage and cell growth arrest due to the action of ATM in the cell cycle. In addition, to compensate for hepatocyte growth arrest, pre-oncogenic cells appeared, with a high rate of proliferation [68]. Therefore, the ATM protein appears to play an important role both in the beginning and progression of NAFLD, including its evolution to HCC.

The main strengths of the present study with A-T subjects include the unprecedent description of a non-invasive diagnosis of liver fibrosis and a comprehensive prospective data collection on several metabolic and inflammatory biomarkers. The absence of genotyping for *ATM* gene variants and the small number of patients with significant hepatic fibrosis are some limitations of the current study. Furthermore, its unicentric design prevented an increase in the sample size, which could conceivably limit the evaluation of potential confounding variables, such as age and time of disease progression.

## Conclusions

Our results showed significant liver fibrosis by non-invasive markers in 20% of A-T patients. The presence of fibrosis was associated with elevation in liver enzymes and serum ferritin, increase in the HOMA-AD, and severity of ataxia. The present study emphasizes the importance of monitoring liver and metabolic changes during follow-up through non-invasive imaging and laboratory methods, especially from adolescence. Additionally, modifiable factors such as inflammation and alterations in glucose metabolism seem to be related to the progression of liver disease, opening new possibilities for the development of future therapeutic interventions.

## Supplementary Information

**Additional file 1: Table S1**. Comparison of the patients with Ataxia-telangiectasia according to the presence or absence of liver steatosis.

**Additional file 2: Fig. S1**. 11-years-longitudinal follow-up of the mean values of the aspartate aminotransferase to platelet ratio. Individualized APRI values of 4 patients with ataxia-telangiectasia with a non-invasive diagnosis of significant hepatic fibrosis.

## Data Availability

All data generated or analysis during this study are included in this published article.

## Abbreviations

ALT: Alanine aminotransferase
ALKP: Alkaline phosphatase
APRI: AST to platelet ratio index
AST: Aspartate aminotransferase
A-T: Ataxia-telangiectasia
ATM: Ataxia telangiectasia mutated
BMI: Body mass index
CAP: Controlled attenuation parameter
CK-18: Cytokeratin-18
MUAC: Mid-upper arm circumference
TC: Total cholesterol
ELISA: Enzyme linked immunosorbent assay
ESID: European society for immunodeficiencies
GGT: Gamma glutamyl transferase
HbA1c: Glycated hemoglobin
HCC: Hepatocellular carcinoma
HDL-c: High density lipoprotein cholesterol
HOMA-AD: Homeostasis model assessment—adiponectin
HOMA-IR: Homeostasis model assessment—insulin resistance
HSC: Hepatic stellate cell
hs-CRP: High-sensitivity C-reactive protein
ICARS: International cooperative ataxia rating scale
IQR: Interquartile range
IR: Insulin resistance
LDL- c: Low density lipoprotein cholesterol
LMS: Liver stiffness measurement
kPa: Kilopascals
KAS: Klockgether ataxia score
NAFLD: Nonalcoholic fatty liver disease
NASH: Nonalcoholic steatohepatitis
NCEP: National cholesterol education program
NFS: Nonalcoholic fatty liver disease fibrosis score
NHDL-c: Non-HDL-c
OGTT: Oral glucose tolerance test
rho: Correlation coefficient
ROS: Reactive oxygen species
SAA: Serum amyloid A
SR: Success rate
TE: Transient elastography
TG: Triglycerides
TNF-α: Tumor necrosis factor alpha
WC: Waist circumference
WHtR: Waist circumference to height ratio
