# Supplementary material for: Hepatic fibrosis: a manifestation of the liver disease evolution in patients with Ataxia-telangiectasia
Source: Orphanet J Rare Dis. 2023 May 5;18:105. doi: 10.1186/s13023-023-02720-7 (PMC10161655; doi:10.1186/s13023-023-02720-7)
Supplement: Supplementary file 1 — Additional file 1: Table S1. Comparison of the patients with Ataxia-telangiectasia according to the presence or absence of liver steatosis. [file 13023_2023_2720_MOESM1_ESM.docx]

**Supplementary Material**

**Table 1.** Comparison of the patients with Ataxia-telangiectasia according to the presence or absence of liver steatosis

| **Variables** | **Unit** | **Absence of liver steatosis**  **(n=7)** | **Presence of liver steatosis**  **(n=13)** | **p-value^a,b^** |  |
| --- | --- | --- | --- | --- | --- |
| **AST** | U/L | 43.6 (16.6-75.5)^c^ | 33.5 (16.6-82.3) | 0.874^a^ |  |
|  |  |  |  |  |  |
| **ALT** | U/L | 30.7 (16.3-66.1) | 29.8 (14.2-144.5) | 0.968^a^ |  |
|  |  |  |  |  |  |
| **GGT** | U/L | 31.0 (7.0-259.0) | 21.0 (11.0-612.0) | 0.843^a^ |  |
|  |  |  |  |  |  |
| **CK-18** | mIU/mL | 287.5 ± 247.5^d^ | 245.8 ±192.0 | 0.680^b^ |  |
|  |  |  |  |  |  |
| **AFP** | UL/mL | 323.9 (127.0-400.0) | 164.4 (74.2-625.0) | 0.405^a^ |  |
|  |  |  |  |  |  |
| **Ferritin** | ng/mL | 99.1 (55.9-415.0) | 94.8 (50.2-1538.0) | 0.501^a^ |  |
|  |  |  |  |  |  |
| **hs-CRP** | mg/L | 1.0 (0.2-53.1) | 1.0 (0.4-13.6) | 0.539^a^ |  |
|  |  |  |  |  |  |
| **TNF-alpha** | pg/mL | 144.7 ± 10.3 | 138.5 ± 10.7 | 0.223^b^ |  |
|  |  |  |  |  |  |
| **Adiponectin** | µg/mL | 7.2 ± 4.9 | 5.9 ± 3.7 | 0.527^b^ |  |
|  |  |  |  |  |  |
| **SAA** | ng/mL | 18.4 (9.0-147.2) | 9.5 (9.0-73.7) | 0.352^a^ |  |
|  |  |  |  |  |  |
| **HOMA-IR** | - | 1.4 (0.08-1.6) | 1.7 (0.08-20.9) | 0.171^a^ |  |
|  |  |  |  |  |  |
| **HOMA-AD** | - | 2.7 (0.2-7.9) | 6.7 (0.5-213.9) | 0.140^a^ |  |
|  |  |  |  |  |  |
| **Matsuda index** | - | 9.3 (4.2-81.3) | 8.0 (0,5-56.0) | 0.282^a^ |  |
|  |  |  |  |  |  |

^a^ Significance Level of the Mann-Whitney U test.

^b^ Significance Level of independent Student

^c^ Median (minimum – maximum)

^d^ Mean (standard deviation)

Abbreviations: ALT (alanine aminotransferase), AST (aspartate (aminotransferase), GGT (gama-glutamyl transferase), CK-18 (cytokeratin-18), AFP (alpha-fetoprotein), hs-CRP (high sensitivity C reactive protein), TNF-alpha (tumor necrosis factor alpha), SAA (serum amyloid A protein), HOMA-IR (Homeostasis Model Assessment - Insulin Resistance) and HOMA-AD (Homeostasis Model Assessment - Adiponectin).
