# Supplementary figures and images for: Hepatic fibrosis: a manifestation of the liver disease evolution in patients with Ataxia-telangiectasia
Source: Orphanet J Rare Dis. 2023 May 5;18:105. doi: 10.1186/s13023-023-02720-7 (PMC10161655; doi:10.1186/s13023-023-02720-7)

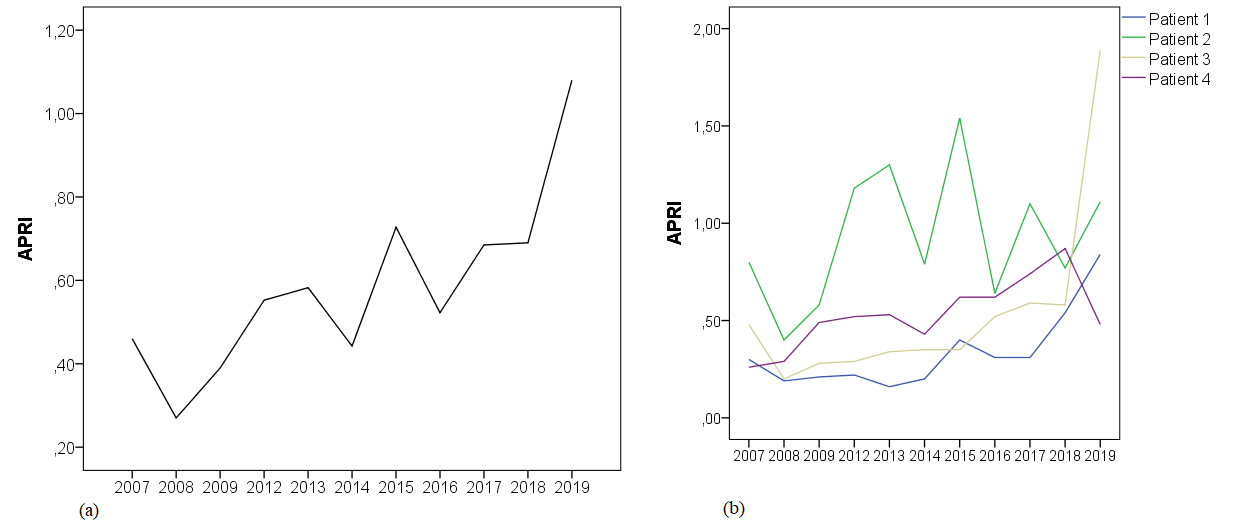

Supplement: Supplementary file 2 — Additional file 2: Fig. S1. 11-years-longitudinal follow-up of the mean values of the aspartate aminotransferase to platelet ratio. Individualized APRI values of 4 patients with ataxia-telangiectasia with a non-invasive diagnosis of significant hepatic fibrosis. [file 13023_2023_2720_MOESM2_ESM.tif]
